# Advances in IoMT for Healthcare Systems

**DOI:** 10.3390/s24010010

**Published:** 2023-12-19

**Authors:** Muhammad Shafiq, Jin-Ghoo Choi, Omar Cheikhrouhou, Habib Hamam

**Affiliations:** 1 Department of Information and Communication Engineering, Yeungnam University, Gyeongsan 38541, Republic of Korea; 2 Higher Institute of Computer Science of Mahdia, University of Monastir, Mahdia 5000, Tunisia; omar.cheikhrouhou@isetsf.rnu.tn; 3 Faculty of Engineering, Université de Moncton, Moncton, NB E1A 3E9, Canada; habib.hamam@umoncton.ca; 4 International Institute of Technology and Management, Avenue des Grandes Ecoles, Libreville P.O. Box 1989, Gabon; 5 School of Electrical Engineering, Department of Electrical and Electronic Engineering Science, University of Johannesburg, Johannesburg 2006, South Africa; 6 Spectrum of Knowledge Production & Skills Development, Sfax 3027, Tunisia

## 1. Introduction

Nowadays, the demand for healthcare to transform from traditional hospital and disease-centered services to smart healthcare and patient-centered services, including the health management, biomedical diagnosis, and remote monitoring of patients with chronic diseases, is growing tremendously. This is due to the facts that traditional healthcare systems are under increasing pressure due to the steady aging of global population and evolving risks of disease outbreaks such as the COVID-19 epidemic [1]. In this regard, the Internet of Medical Things (IoMT) has significant potential, which involves multiple disciplines (including informatics, computer science, bioengineering, etc.) contributing to the design of more responsive and efficient healthcare systems. The rapid growth of micro-computing devices, data-processing algorithms, and supporting communication protocols can enable new Internet of Things (IoT) solutions to reshape IoMT applications because they carry tremendous ability to gather, analyze, and share big data on the Internet. However, the advances in IoMT technologies face numerous daunting challenges due to their multidisciplinary nature and rapidly growing demands in cyber–physical health management, big data collection and analysis, therapeutic approaches, medication management, wearable and flexible devices, biosensor quality and user experience, etc. [2]. The key research areas in IoMT are outlined as follows.

### 1.1. Standards for Interoperability

The lack of standards for healthcare interoperability is one of the major barriers to IoMT implementation. In the IoMT environment, multiple entities such as users, devices, and information sources participate in providing healthcare services [3]. For example, remote patient monitoring requires a variety of personal health devices, data processing algorithms, and communication protocols that include the measuring, processing, and transmitting of vital signs. The collected data are converted into valuable information and sent to the cloud server via the gateway node. For healthcare, doctors and caregivers can remotely access the stored information from the cloud by reducing data soils. This process appears simple, but interoperability becomes a significant challenge when it comes to the implementation of internetworking across different types of devices, information sources, and platforms. Without the common standards in healthcare, we cannot accomplish the benefits of seamless communication under diverse needs of IoMT solutions. In this context, ANSI HL7 International established a series of standards [4] which are of great interest in view of the growing needs of IoMT for various specifications of interoperability, such as electronic health records; personal health record; exchange of medical documents and resources; structured product labeling; and representation of clinical summaries, medical recommendations, and clinical conditions.

### 1.2. Security and Privacy of Helthcare Data

IoMT services must address the bring-your-own-device (BYOD) phenomenon, where patients and doctors use their personal devices to access medical data from the cloud. On the one hand, the use of BYOD policies makes IoMT services (such as e-visits, telemedicine, and remote patient monitoring) crowdsourced and cost-effective. On the other hand, it also brings major challenges to the security and protection of medical data. Therefore, we need to robustly deploy security and privacy controls (e.g., NIST SP800-30, ISO 27001, ISO/IEC Standard 27018 [5,6]) to protect medical data prevalent on the Internet from becoming prey to hackers. In this context, many research topics have received attention, including, but not limited to, the following: risk assessment, device security, session security, cloud security, data protection, and authentication.

### 1.3. Wearable Healthcare Devices and Edge Computing

The use of wearable devices has become an important part of IoMT services for the remote measurement and continuous tracking of patients’ vital signs. These devices can easily be used as accessories, integrated into clothing, applied to the skin as tattoos, or even embedded into the bodies of older adults or those with chronic illnesses when worn. Despite the many advantages of wearable devices, the conventional cloud computing systems are under increasing strain due to the influx of their data generation on a large scale. On the other hand, edge computing stands out as a candidate solution to the issues of the increased data volumes. In this regard, the benefits of fast data processing can be achieved along with the improved data security, since in edge computing, data analysis occurs closer to the source rather than sending it to the cloud at a remote location. However, wearable devices in edge infrastructure have certain limitations due to their resource constraints, such as high cost, short battery life, limited functionality and processing power, lower interoperability, and higher data security and privacy risks [7]. Additionally, managing distributed devices increases orchestration complexity in edge infrastructure. As a result, more sophisticated solutions are needed to address many of these challenges and incorporate edge computing as an integral part of IoMT infrastructure.

### 1.4. Machine Learning (ML)- and Artificial Intelligence (AI)-Driven Diagnostics

The integration of machine learning (ML) and artificial intelligence (AI) is becoming increasingly important in the IoMT environment for several reasons. For example, ML and AI can aid in early detection, as such models can quickly analyze large amounts of medical data. ML and AI-driven diagnostics are error-prone and insightful, allowing for more effective and efficient decision-making. Medical robots also help allow us to streamline high-risk clinical tasks such as surgery and radiation therapy [8]. These technologies can predict disease progression and outcomes based on historical data patterns and facilitate real-time data collection and analysis, such as telemedicine and remote patient monitoring. However, the accuracy of ML- and AI-driven applications heavily depends on the quality of the training data, so bias in the training or learning data can lead to unreliable results. In addition, ML models are prone to overfitting or underfitting problems. The successful implementation of ML- and Al-driven applications in the IoMT environment is also hampered by the collaboration issue between machine and human. 

### 1.5. Smart Hospitals and Mobile Health Services

The continued availability of healthcare services and the sustainability of medical facilities, even under extreme climatic conditions and natural disasters such as earthquakes, hurricanes or floods, is among the main goals of modern healthcare systems [9,10]. The installation of smart hospitals and mobile health services can control the downtime of medical services and provide better care to victims with their mobile health services and always-available infrastructure. In this regard, emergency services require upgrading via IoMT devices and mobile health service integration for virtual consultation and immediate treatment in disaster situations. Additionally, traditional hospitals need to be upgraded by supporting IoT technologies to improve medical responses and better decision-making during emergencies. The use of 5G infrastructure and Wi-Fi 6 technology can enable faster and more reliable communication between IoMT devices, supporting high-speed bandwidth requirements and real-time medical applications.

## 2. Review of Published Articles

Here, we provided a brief overview of the published articles (or contributions). 

In Contribution 1 of this Special Issue, the authors proposed a biometric framework that involves human finger–knuckle print (FKP) and iris for the authentication of users. The authors used biometric fusion of multiple images including the outer surface image of FKP finger phalangeal joint and skin pattern image of the iris. The pattern matching of the obtained images is performed at a match score level, in which the neuro-fuzzy classifier is used for features extraction and anomaly detection. The accuracy is reported as greater than 98%, which is higher than that of single biometric classifiers. The use of multiple biometrics is attributed as a solid recognition system in terms of biometric fusion of inherent physical and/or behavioral characteristics compared to a single biometric such as the fingerprint, face, palm print, retina, voice, hand geometry, gait, etc. 

In Contribution 2, the authors employed the deep learning model of the convolutional neural network (CNN) for the initial stage detection and classification of Alzheimer ’s disease (AD), which is a type of brain disorder and dementia. Neuroimaging modalities are used to capture brain modifications through PET (positron emission tomography) and categorize the AD patterns in terms of normal, mild, and severe. The CNN architectures are deployed in both 2D and 3D domains, in which blurring filters have been used before the downside subsampling of the images. The 2D and 3D CNN architecture outperformed the existing classification methods in terms of accuracy. The use of modern ML models not only helps diagnose and classify AD, but can also be used for the early detection of other fatal diseases. In Contribution 3, the authors used AI and hybrid deep learning algorithms for optimized fetal health records in cardiotocography (CTG), which is a composite diagnostic test of the fetal uterine activity and heart rate. In this research, a deep transfer learning model is proposed to reduce computational time and optimize classification accuracy in CTG records. The proposed model achieved better results compared to the related models (including: GoogleNet, DenseNet, RCNNs, and ResNet) in a real-time clinical environment. In modern clinical practices, good classification specificity and accuracy rate of CTG datasets can enable informed decision-making and a timely referral of chronic complications during delivery. 

In Contribution 4, the authors focused on the implementation of blockchain technology with novel architecture to protect healthcare data in cyberspace. In this architecture, the network members are distributed into decentralized clusters while a single copy of the blockchain is preserved for each cluster. The proposed architecture offers privacy protection and data security against cyberattacks with low computational and communication overhead compared to bitcoin and lightweight blockchain networks. The design of novel blockchain data security algorithms is more crucial for patients’ trust because cyber-attacks are becoming more diverse with the growth of network nodes. In this regard, the proposed architecture could be a good candidate solution because it can reduce network traffic and improve ledger updates with the increase in the number of nodes. Contribution 5 discusses the implementation of deep learning models for object detection and face tracking in an automatic invigilation system. The training and testing accuracies of the suggested models are reported as good. The proposed invigilation model can equally be used for the provision of smart healthcare services such as remote patient monitoring and telemedicine. 

In Contribution 6, the authors present the analysis of network slicing (NS) regarding the increasing commercial needs and developing network challenges of fifth-generation (5G) technology. The 5G operators can offer customized network capacity and local services according to the requirements of their clients by using the use-case of network slicing. The operators can also establish a strategic viewpoint of their costs and revenues regarding actors, functions, architectures, and processes during the slicing of their networks. Furthermore, the service providers can unlock the core benefits of AI and ML in various standards and industrial forums to incorporate intelligence and automation in their network services. The suggested NS can help design healthcare models that are agility smart, more efficient, and cost-effective. 

In Contribution 7, the authors conducted a detailed review of IoT-enabled data acquisition of human vital signs through non-invasive mechanisms that do not require skin contact. It is revealed that health informatics is lacking the applications of a self-power high-speed smart processing unit that can perform real-time non-invasive data acquisition. These processing units should be additionally powered with the cross-platform ability to communicate with different nodes. In this regard, the synergistic integration of modern technologies (i.e., AI, deep learning, and metaverse, etc.) is required to empower the sensing techniques and multi-camera systems. In Contribution 8, a cooperative energy-efficient routing protocol is proposed for underwater wireless sensor networks. Therein, the hotspot issue is resolved with the help of sink mobility. The sensor nodes collaboratively share their data with the sink nodes to increase the network lifetime and harvest energy. In mobile healthcare, applications of energy-efficient protocols are of much interest for online patient monitoring and data acquisition tasks.

In Contribution 9, the authors proposed an adaptive support vector machine (SVM) classifier for the improved classification of electroencephalography (EEG) signals. The traditional common spatial patterns (CSP) filtering method has shortcomings relating artifacts, whereby the ECG components are distorted. The proposed feature based adaptive extraction method outperforms the existing methods in terms of information transfer rate and classification accuracy. In Contribution 10, the authors proposed a deep learning based novel method for emotion detection through multimodal physiological signals. For emotion charting, the accurate classification of physiological signals is challenging due to their non-linear nature and noise enclosure during their recording. The proposed method processes the physiological signals to remove noise with different filtering, classification and deep learning methods for the identification of four different emotional states. The accuracy of the proposed system is reported higher than that of the other related methods. In IoMT, such agility smart emotion charting methods are of great demand especially for stroke-affected patients and therapeutic treatments.

In Contribution 11, the authors proposed an improved fall detection model aimed at elderly individuals. It is a hardware-independent model that uses transfer learning on small datasets to make it generalizable for various wearable devices. The results showed that the proposed transfer learning model has considerably improved the performance in terms of the area under the curve, F1-scores, and false positive prediction rate for fall detection. We need sophisticated fall detection systems because major falls cause morbidity and mortality, especially in older adults. In Contribution 12, the authors proposed an interactive system for cardiovascular diseases. The system collects and analyzes cardiovascular data over the Internet using preprocessing algorithms and mathematical analysis of cardiac records to ensure continuous healthcare, especially for patients in remote areas. In Contribution 13, we found a monitoring system that can support ML, AI, and IoT solutions to ensure more accurate healthcare treatment for heart failure patients. In Contribution 14, the authors used deep learning and AI techniques to detect masks, an often-neglected area of research in IoMT prior to the COVID-19 pandemic. The proposed system provides an excellent opportunity for real-time monitoring and identification of mask protocol violations in e-governments. 

In this Special Issue, we have been constantly looking for developing IoMT solutions and novel applications for healthcare systems. We followed a rigorous review process to select the following 14 published articles from the 30 received papers.

## 3. Conclusions

In this Special Issue, the authors mainly focused on the integration of ML, AI, 5G, and IoT technologies in healthcare research. However, the pace of successful integration and implementation of promising IoT technologies is still slow because data security and privacy, transfer learning, and interoperability issues of medical applications are challenging. Moreover, the integration of IoT technologies in healthcare is an ongoing research area due to a number of issues such as non-invasive data acquisition, emotion recognition, better disease diagnosis and medical error, biomedical big data analytics, accuracy of wearable sensors, and health surveillance of patients in remote settlements. Therefore, sophisticated solutions are needed to address many of these challenges and accelerate the development of patient-centric mobile services and smart hospitals through the successful integration of 5G networks, IoT, and computing technologies.

## References

[1] Baker S., Xiang W. (2023). Artificial Intelligence of Things for Smarter Healthcare: A Survey of Advancements, Challenges, and Opportunities. IEEE Commun. Surv. Tutor..

[2] Osama M., Ateya A.A., Sayed M.S., Hammad M., Pławiak P., Abd El-Latif A.A., Elsayed R.A. (2023). Internet of medical things and healthcare 4.0: Trends, requirements, challenges, and research directions. Sensors.

[3] Lee E., Seo Y.D., Oh S.R., Kim Y.G. (2021). A Survey on Standards for Interoperability and Security in the Internet of Things. IEEE Commun. Surv. Tutor..

[4] Koncar M. (2005). HL7 standard—Features, principles, and methodology. Acta Medica Croat. Cas. Hravatske Akad. Med. Znan..

[5] Kitsios F., Chatzidimitriou E., Kamariotou M. (2023). The ISO/IEC 27001 Information Security Management Standard: How to Extract Value from Data in the IT Sector. Sustainability.

[6] Cuevas-González D., García-Vázquez J.P., Bravo-Zanoguera M., López-Avitia R., Reyna M.A., Zermeño-Campos N.A., González-Ramírez M.L. (2022). ECG Standards and Formats for Interoperability between mHealth and Healthcare Information Systems: A Scoping Review. Int. J. Environ. Res. Public Health.

[7] Shanmugam B., Azam S. (2023). Risk Assessment of Heterogeneous IoMT Devices: A Review. Technologies.

[8] Ahmed I., Jeon G., Piccialli F. (2022). From artificial intelligence to explainable artificial intelligence in industry 4.0: A survey on what, how, and where. IEEE Trans. Ind. Inform..

[9] Kumar A., Nanthaamornphong A., Selvi R., Venkatesh J., Alsharif M.H., Uthansakul P., Uthansakul M. (2023). Evaluation of 5G techniques affecting the deployment of smart hospital infrastructure: Understanding 5G, AI and IoT role in smart hospital. Alex. Eng. J..

[10] Kwon H., An S., Lee H.Y., Cha W.C., Kim S., Cho M., Kong H.J. (2022). Review of smart hospital services in real healthcare environments. Healthc. Inform. Res..

